# Woods’s Medical and Surgical Monographs

**Published:** 1889-11

**Authors:** 


**Wood's Medical and Surgical Monographs.** Vol. IV., No. 1. October, 1889. New York: William Wood & Company, 56 and 58 Lafayette place. Published monthly. Price, $10.00 a year; single copies, $1.00.

The contents of the current number of this interesting work embraces the following titles: The Influence of the Male Element upon the Female Organism, by John Brown, M. D.; The Internal and External Temperature of the Human Body as Modified by Muscle-Kneading, by A. Symons Eccles, M. B.; The Disease of the Breast, by Thomas Bryant, F. R. C. S.

The first essay was read before the Glasgow Southern Medical Society, March 8, 1888, and is an interesting study of this abstruse question. The views of the author are fortified by observations of Darwin and others, in experiments upon plants and animals. His conclusions are "that the male element has an influence upon the female, over and above its fertilizing influence upon the ovum. . . The limits of that influence are at present unknown. The wide range suggested by Dr. Harvey (fetal inoculation) is by no means proven, but is quite probable; the evidence does not entitle us to go beyond this."

The subject of the second essay, read in the Section of Anatomy and Physiology, at the annual meeting of the British Medical Association, held in Glasgow, August, 1888, is a novel one, and will prove interesting in connection with the therapeutic application of massage.

The bulk of this number, 288 pages, is devoted to Diseases of the Breast, and is illustrated with thirteen engravings and four chromo-lithographs. It is by far the most exhaustive treatise of the subject that has yet appeared in literature, and is well worthy its distinguished author. The name of Thomas Bryant is a guaranty of meritorious and instructive work in any field that he consents to enter with his pen, and he never wields it with an aimless purpose. When Bryant writes, it is because he has something to say, and he says it with such scholarly grace and conciseness, that it makes delightful as well as helpful reading.

The interest in this valuable series is well maintained in the present number, and it should meet with increased patronage.

